# Evaluating the Impact of Reproductive and Sexual Health Education on Knowledge and Attitudes of Couples in Premarital Counseling Classes: A Quasi‐Experimental Study

**DOI:** 10.1002/hsr2.71921

**Published:** 2026-02-28

**Authors:** Elnaz Ashrafi, Leila Baeiman Oskoei, Mahasti Emami Hamzehkolaee, Bahare Izadi, Morteza Mansourian, Behrouz Bahadori

**Affiliations:** ^1^ Department of Health Education and Promotion School of Health, Guilan University of Medical Sciences Rasht Iran; ^2^ Health Promotion Research Center Iran University of Medical Sciences Tehran Iran; ^3^ PHD of Health Education and Promotion, Clinical Research Development Unit of Shahid Yahyanejad Hospital Babol University of Medical Sciences Babol Iran

**Keywords:** attitude, knowledge, reproductive health, sexual health

## Abstract

**Background and Aims:**

Educating couples about reproductive and sexual health is crucial for enhancing marital well‐being, fostering healthy sexual relationships, and ultimately strengthening family bonds. The purpose of this study is to determine the impact of reproductive and sexual health education on knowledge and attitudes of couples in premarital counseling classes.

**Methods:**

This quasi‐experimental controlled intervention study involved 200 people (100 couples) attending the General Health Service Center No. 8 in Rasht city. Forty eligible couples were selected using simple random sampling and randomly assigned to two groups: the intervention group (20 couples) and the control group (20 couples). Data were collected using a researcher‐designed questionnaire comprising two sections: the first covered four demographic questions, while the second assessed couples' knowledge and attitudes toward reproductive and sexual health. The intervention group participated in a tailored reproductive and sexual health education program delivered five 60‐min sessions. Questionnaires were administered before the intervention and 3 months afterward. Data were analyzed using SPSS version 24, employing descriptive statistics, Chi‐square tests, Mann–Whitney *U* test, and Wilcoxon tests with a significance level of *p* < 0.05.

**Results:**

Demographic variables were homogeneous across both groups, with no statistically significant differences observed (*p* > 0.05). Prior to the intervention, there was no significant difference in the average knowledge and attitude scores between the intervention and control groups. However, following the educational program, the intervention group demonstrated a significant increase in these scores (*p* < 0.0001).

**Conclusion:**

The findings underscore the effectiveness of premarital educational interventions in enhancing couples' knowledge and attitudes regarding reproductive and sexual health.

## 1 Introduction

Marriage and the establishment of a marital relationship are among the most significant interpersonal actions, providing psychological benefits such as trust, commitment, and satisfaction [1]. Healthy sexual relationships between couples strengthen family bonds, decrease the risk of mental health issues, and help maintain the stability of both the family unit and society as a whole [2]. Sexual health is a critical component of individual well‐being, serving as the foundation of physical, emotional, and psychological health for individuals, couples, and families. It affects people of all ages and life stages, playing a vital role in the overall health and harmony of families. Moreover, it has been recognized as a fundamental human need and a strategy for achieving the Millennium Development Goals [3].

Sexual health is defined as a state in which couples engage in healthy, appropriate, and fulfilling sexual relationships, reflecting harmony, intimacy, and love within their marital lives [4]. This requires favorable physical, mental, and behavioral conditions. It is considered a fundamental right of both men and women to be prepared for their sexual responsibilities and acquire the necessary knowledge and awareness regarding sexual health [5]. Research indicates that although 40% of couples report satisfaction with their marriage, many still experience sexual dysfunction or dissatisfaction in their sexual relationships [6]. Furthermore, approximately 50% of married women over the age of 35 have never experienced orgasm, while 30%–40% of men seeking treatment for sexual dysfunction report premature ejaculation [7]. Adolescence is a critical developmental stage, making the reproductive health of adolescents and young people a global priority. It is regarded as a cornerstone for ensuring future societal well‐being and fostering sustainable national development [8]. Reproductive health encompasses a state of complete physical, mental, and social well‐being in all matters related to the reproductive system, its processes, and functions throughout an individual's life [9].

Educating couples about reproductive health and sexual matters plays a crucial role in promoting marital well‐being, fostering healthy sexual relationships, and ultimately strengthening the family unit. Currently, premarital education programs focuses on essential topics such as reproductive health services, childbearing, and family stability through educational, therapeutic, and preventive approaches [10]. Premarital classes provide couples with the opportunity to understand their mutual and individual rights and responsibilities regarding reproductive health. Participation in these classes can lead to significant positive changes in attitudes, further supporting informed and healthier relationships [11].

Recent studies have highlighted the effectiveness of various educational methods in premarital counseling. For example, Bahrami‐Samani et al. compared booklet‐based with video‐based premarital education and found that while both methods significantly improved reproductive health literacy among engaged couples, the video‐based approach had a greater impact on certain aspects of health literacy [12]. Similarly, Izadi et al. [13] developed a sexual health education package and demonstrated its positive impact on sexual functioning among newly married couples.

Rasht, the capital city of Gilan Province, is characterized by a notably aging population and a declining fertility rate, with recent studies reporting fertility rates below the replacement level. For instance, a study conducted among married women aged 15–49 in Rasht found that fertility rates have declined significantly in recent years, reflecting broader demographic trends in the region [14]. This demographic profile highlights the urgent need for targeted reproductive and sexual health education initiatives tailored to the local population to improve knowledge and attitudes, thereby supporting healthier marriages and potentially mitigating demographic challenges [15].

Therefore, considering the critical role of knowledge and positive attitudes in establishing healthy marriages and addressing regional demographic challenges, the present study aimed to determine the impact of reproductive and sexual health education on the knowledge and attitudes of couples participating in premarital counseling programs.

## 2 Materials and Methods

This quasi‐experimental, controlled interventional study was conducted on couples attending premarital counseling classes in 2023. The study design followed standard recommendations for quasi‐experimental research [16]. Inclusion criteria were being in a first‐time marriage, holding Iranian nationality, having basic literacy, and expressing a willingness to participate. Exclusion criteria were withdrawal from participation or missing more than one training session.

The sample size was determined using the Cochran formula, based on a population of 200 individuals (100 couples) attending a general health service center. The calculations assumed an estimation precision (*d*) of 0.1, a percent homogeneity of responses (*p*) of 50%, a normal distribution value (*z*) of 1.96, and a significance level (*α*) of 0.05, resulting in a required sample size of 80 participants (40 couples). The Cochran formula used is as follows:$n0=(Z^{2}\times p\times (1‐p))/d^{2}.$

Based on this calculation, 40 eligible couples (80 participants) were randomly selected from the accessible population of 100 couples at General Health Service Center No. 8 in Rasht City, and then randomly assigned to either the intervention group (20 couples) or the control group (20 couples). This approach ensured that the study met the calculated statistical requirements while maintaining representativeness of the population.

Data were collected using a two‐part questionnaire developed by the research team. The first section gathered demographic information, including age, education level, occupation, and place of residence. The second section assessed participants' knowledge and attitudes regarding sexual health and fertility. Awareness was measured using 12 multiple‐choice questions, scored on a scale of 0 to 3, with total scores ranging from 0 to 36. Attitudes were measured using 12 questions based on a 5‐point Likert scale, with a total score range of 12–60.

The content validity of the questionnaire was assessed by a panel of 10 reproductive health professionals and 5 health education and promotion professionals. The content validity coefficient (CVR) was calculated as 0.83 and the content validity index (CVI) determined to be 0.86. Reliability was assessed using internal consistency. The questionnaire was administered to 20 couples (not included in the study sample) and the Cronbach's alpha coefficient was 0.71 for the knowledge section and 0.73 for the attitude section.

After obtaining necessary permissions and ethical approval, the researcher approached General Health Service Center No. 8 in Rasht and selected 40 eligible couples from 100 couples through random sampling. These participants were then divided into two groups, the intervention group and the control group, each consisting of 20 couples, using simple random sampling. Study participants were thoroughly informed about the study process, confidentiality of information, and the purpose of the study. Written informed consent was obtained from all participants. Both the intervention and control groups completed an initial questionnaire. The intervention group participated in an educational program that consisted of five 60‐min sessions. The educational content covered key topics such as maintaining a stable and happy family, familiarity with the sexual organs, sexual function, reproductive health, special care during the reproductive period, high‐risk pregnancies and their complications, contraceptive methods, and common sexually transmitted diseases. Meanwhile, the control group did not receive any educational intervention. Three months after the end of the educational program, the questionnaires were redistributed and completed by the participants in both groups. The data were entered into SPSS version 24.0 and analyzed using descriptive statistics (median and interquartile range), Chi‐square tests, Mann–Whitney *U* test, and Wilcoxon tests, with a significance level set at *p* < 0.05. The Kolmogorov–Smirnov test was applied to assess the normality of the data distribution, which indicated non‐normal distribution. Therefore, non‐parametric tests were employed as appropriate alternatives to provide more reliable results given the data characteristics [17].

### 2.1 Findings

In this study, the mean age of the intervention group participants was 27.95 ± 5.88 years, and the mean age of the control group participants was 27.23 ± 4.71 years. The gender distribution was the same. Fifty percent were female participants and 50% were male participants. As shown in Table 1, the intervention and control groups were similar in demographic variables, and there was no statistically significant difference (*p* > 0.05). Table 2 shows that before the educational intervention, there was no statistically significant difference in the mean scores of knowledge and attitudes about sexual and reproductive health between the two groups. However, after the educational program, the mean scores of these variables in the intervention group increased significantly (*p* < 0.0001).

**Table 1:** Demographic comparison of couples in intervention and control groups.

Variables	Control (*n* = 40)	Intervention (*n* = 40)	*p* value^a^
Employment status			
Unemployed	3 (%7.5)	2 (%5)	0.66
Housewife	9 (%23.5)	11 (%27.5)	
Employee	11 (%27.5)	10 (%25)	
Worker	3 (%7.5)	4 (%10)	
Freelance	14 (%25)	13 (%32.5)	
Location			
City	36 (%90)	34 (%85)	0.15
Village	4 (%10)	6 (%15)	
Educational level			
Undergraduate	3 (%7.5)	5 (%12.5)	0.24
Diploma	8 (%20)	6 (%15)	
University	29 (%73.5)	29 (%73.5)	

*Note:* Values are presented as number and present.

^a^ Chi‐square test was used.

**Table 2:** Comparison of knowledge and attitude on sexual and reproductive health among couples in intervention and control groups.

Variable /Time	Control (*n* = 40) Median [IQR]	Intervention (*n* = 40) Median [IQR]	*p* value^a^
Knowledge			
Pre‐intervention	19 [5]	18 [6]	0.26
Post‐intervention	20 [4.25]	29 [4]	0.0001
Effect size (*r*)^b^	0.05	0.62	
*p* value^c^	0.4	0.0001	
Attitude			
Pre‐intervention	49 [8.75]	50 [11]	0.52
Post‐intervention	51 [11]	58 [10.25]	0.0001
Effect size (*r*)^b^	0.07	0.57	
*p* value^c^	0.38	0.0001	

Abbreviation: IQR, interquartile range.

^a^ Mann–Whitney *U* test was used.

^b^ Calculated as *Z*/√*N*; applicable for non‐parametric tests.

^c^ Wilcoxon tests test was used.

## 3 Discussion

The purpose of this study was to evaluate the impact of reproductive and sexual health education on the knowledge and attitudes of couples participating in premarital counseling classes. The intervention group showed a significant increase in knowledge scores compared to the control group following the educational program. These results are consistent with those of Kohn et al. [18], Leekuan et al. [19], Izadi et al. [13], Khadivzadeh et al. [20], Ramazani et al. [21], and Torkian et al. [22] who demonstrated the positive impact of reproductive and sexual health education on participants' knowledge, attitudes, and behaviors. One of the key factors contributing to the success of the program was the structured format of the premarital counseling sessions. The purpose of these sessions was to enhance participants' knowledge of various sexual and reproductive health topics and to promote positive attitudes, thereby laying the foundation for a successful marriage [23].

The curriculum covered important areas such as an introduction to anatomy, sexual function, reproductive health, special care during pregnancy, high‐risk pregnancies and their complications, contraceptive methods, and sexually transmitted disease. Using effective teaching techniques such as delivering key messages for a stable and happy family life, providing sufficient Q&A time, encouraging group discussions and brainstorming, and utilizing the instructor's teaching methods played a key role in enhancing participants' awareness. The premarital education also emphasized topics such as contraception, appropriate child spacing, optimal age for conception, and family size planning according to the couple's situation. Unplanned pregnancies without adequate economic, social, and psychological preparation can disrupt family stability. Research has shown that increased knowledge can lead to positive changes in attitudes [10]. Therefore, sexual and reproductive health education is essential because appropriate knowledge promotes healthy attitudes and responsible behaviors [24].

Communication barriers, unexpressed needs, false beliefs, and incorrect attitudes—especially among women—pose significant challenges for young couples. Cultural norms emphasizing tolerance, neglect of women's sexual health, and shame around discussing sexual issues often perpetuate cycles of unsatisfactory sexual relationships [25]. Addressing these issues in premarital education by teaching communication skills, debunking false beliefs, and promoting accurate information proved effective in shaping couples' attitudes. Additionally, the presence of both partners in these sessions was instrumental in influencing attitudes and social norms. Active participation of spouses served as a persuasive factor, reinforcing the objectives of the program [26]. Therefore, comprehensive health programs focusing on reproductive and sexual health are essential for ensuring the well‐being of families and society at large.

In the control group, while the level of awareness and attitudes among couples showed a slight increase, this change was not statistically significant. This finding aligns with studies conducted by Ranjbar et al. [27] and Izadi et al. [13]. The observed rise in awareness and attitude scores in the control group may be attributed to the mandatory participation of couples in premarital counseling sessions. Despite the existence of these obligatory classes and the World Health Organization's emphasis on sexual health education as a fundamental human right and a development necessity, Iran still lacks a comprehensive national program for sexual health education. Consequently, many needs of young couples in this area remain unmet [28], and only a limited number of interventions have been conducted in the field of sexual education and counseling for couples preparing for marriage [29].

The primary goal of education is to enhance learners' awareness to facilitate behavioral change. Therefore, it is recommended to focus on improving the quality of educational classes by tailoring content to the the specific needs of couples. These sessions should incorporate effective teaching methods, suitable educational aids, appropriate venues and schedules, and experienced instructors.

## 4 Conclusion

The results of this study demonstrate that educational interventions on reproductive and sexual health are effective in increasing the awareness and attitudes of couples attending premarital counseling sessions. Marriage represents an important new stage in life, and sexual health education is essential during this transition. To promote stable and healthy marital relationships, young people need comprehensive knowledge about reproductive health, especially sexual relations. Developing premarital education programs that meet the identified needs of couples is a key strategy for meeting these needs.

The findings highlight the importance of integrating reproductive and sexual health education into premarital counseling programs on a broader scale. These interventions can be scaled up to other health centers and regions, especially in areas with low fertility rates and aging populations. Policymakers should consider supporting the expansion of evidence‐based premarital education programs, allocating resources for training health professionals, and developing culturally sensitive educational materials. Such efforts can contribute to improved marital health, reduced sexual dysfunction, and enhanced family stability at the community and national levels.

## 5 Limitations

One limitation of this study is the reliance on self‐reported data, which may have introduced response bias in the survey. Additionally, the moderate internal consistency of the measurement instruments, as indicated by Cronbach's alpha values ranging from 0.71 to 0.73, suggests some variability in reliability that could have influenced the precision of the results. Future studies should aim to improve the reliability of these measures to strengthen the findings. Furthermore, the sample was drawn from a single health center in Rasht, a region with distinct cultural and socioeconomic characteristics. These regional and cultural factors may influence participants' attitudes and behaviors, potentially limiting the generalizability of the findings to populations with different backgrounds. Therefore, caution is advised when generalizing the results beyond this setting, and future research should include more diverse samples from multiple regions to enhance generalizability. The lack of long‐term follow‐up to assess the sustainability of the intervention effects. This limits our understanding of how lasting the observed changes are over time. Cultural barriers in discussing sexual health may have restricted open communication and influenced participants' willingness to fully disclose their attitudes and behaviors. In societies with strong cultural taboos, discussing sexual matters can be associated with shame and discomfort, which may further affect reporting accuracy.

## Author Contributions

**Elnaz Ashrafi, Morteza Mansourian, Bahare Izadi:** conceptualization, writing. **Leila Baeiman Oskoei:** literature review, writing assistance. **Morteza Mansourian, Behrouz Bahadori:** statistical analysis, data interpretation. **Leila Baeiman Oskoei, Morteza Mansourian:** results interpretation. **Behrouz Bahadori, Morteza Mansourian:** data collection, coordination. **Elnaz Ashrafi, Mahasti Emami Hamzehkolaee:** supervision, revisions all authors approved final manuscript.

## Funding

The authors received no specific funding for this work.

## Ethics Statement

This study was conducted in accordance with the ethical standards of the Declaration of Helsinki and was approved by the Iran University of Medical Sciences (IR.IUMS.REC.1401.565). All participants read and signed the informed consent form.

## Consent

The authors have nothing to report.

## Conflicts of Interest

The authors declare no conflicts of interest.

## Transparency Statement

The lead author Behrouz Bahadori affirms that this manuscript is an honest, accurate, and transparent account of the study being reported; that no important aspects of the study have been omitted; and that any discrepancies from the study as planned (and, if relevant, registered) have been explained.

## Data Availability

The datasets used and/or analyzed during the current study are available from the corresponding author on reasonable request.
